# Climatic, environmental, and social factors in Visceral Leishmaniasis: A spatio-temporal perspective in Brazilian biomes

**DOI:** 10.1371/journal.pntd.0013842

**Published:** 2025-12-30

**Authors:** Maíra G. Kersul, Lucas Edel Donato, Alexandre Guerra dos Santos, Rafaella Albuquerque e Silva, Marcia Leite de Sousa-Gomes, Fredy Galvis-Ovallos, Vitor Vieira Vasconcelos, Anaiá da Paixão Sevá

**Affiliations:** 1 State University of Santa Cruz, Ilhéus, Bahia, Brazil; 2 Health and Environment Surveillance Secretariat/ Ministry of Health, Brasília, Federal District, Brazil; 3 University Center of Brasília – UNICEUB, Brasília, Federal District, Brazil; 4 Independent Veterinary Practitioner, São Paulo, Brazil; 5 School of Public Health, University of São Paulo – USP, São Paulo, Brazil; 6 Center for Engineering, Modeling and Applied Social Sciences – Federal University of ABC, Santo André, São Paulo, Brazil; London School of Hygiene and Tropical Medicine, UNITED KINGDOM OF GREAT BRITAIN AND NORTHERN IRELAND

## Abstract

Vector-borne diseases (VBDs) remain a major global public health challenge, with considerable economic and social impacts. Phlebotomine sand flies, the vectors of the protozoan responsible for visceral leishmaniasis (VL), are highly sensitive to climatic and environmental conditions, which define biome characteristics. Brazil has six distinct biomes, offering a unique setting to explore the spatial variability of VL. We identified and compared climatic, environmental, and social variables associated with VL incidence (IncVL) across Brazilian municipalities and within each biome, excluding Pampa and Pantanal due to insufficient analytical robustness. The dependent variable was the triennial cumulative incidence of human VL (2008–2022). Independent variables included climatic factors (temperature mean and range, rainfall, and humidity), environmental factors (vegetation cover, land use, and urban density), and social indicators (illiteracy index, uncollected waste, and unconnected sewage system). To assess spatial dependence and the role of ecotone areas, we calculated univariate Global and Local Moran’s I indices. Linear Mixed Models (LMMs) were then fitted for each biome and for Brazil overall, including municipality and time as random effects and accounting for spatial correlation (using R software). Nationally, IncVL was positively associated with mean temperature, savannah & grassland, and uncollected waste, and negatively associated with the illiteracy index. Each biome exhibited distinct relationships. In the Caatinga, IncVL was positively associated with forest formation, urban growth, and humidity range, and negatively with the illiteracy index and agriculture. In the Cerrado, it was negatively associated with agriculture and urban population. In the Amazon, it was positively associated with mean temperature and savannah & grassland, and negatively with mean humidity, deforestation, agriculture, and urban population density. In the Atlantic Forest, it was positively associated with mean temperature and savannah & grassland. These findings demonstrate that VL transmission patterns and their determining factors vary substantially across Brazilian biomes, underscoring the importance of region-specific surveillance and control strategies.

## Author summary

Visceral leishmaniasis (VL) is a neglected disease that affects thousands of people in Brazil and worldwide. Its transmission is strongly influenced by environmental and social conditions, yet how these factors interact to shape disease risk remains poorly understood, especially in a country as large and ecologically diverse as Brazil. In this study, we examined how climatic, environmental, and social factors relate to VL incidence across Brazil’s biomes. Our results showed that each biome exhibits distinct patterns of disease occurrence and risk factors. For instance, agricultural land use was associated with fewer cases of VL, while higher temperatures and areas with lower tree density and greater shrub cover (such as savannahs and grasslands) were linked to increased incidence in certain biomes. Many high-incidence areas were located in transition zones between biomes, where environmental conditions change gradually and vectors and hosts may adapt more easily. Our findings provide new insights into how VL behaves in different ecological settings and highlight the need for targeted, region-specific public health strategies to improve prevention and control of this disease.

## 1. Introduction

Leishmaniases are parasitic diseases caused by protozoa transmitted by sand fly vectors to humans [1,2] and to several mammalian hosts, including domestic and wild canids, marsupials and rodents [3–5]. Among its clinical forms, visceral leishmaniasis (VL) is the most severe, characterized by systemic manifestations and a case-fatality rate of 90–95% when left untreated [6,7]. Globally, an estimated 50,000–90,000 new VL cases occur each year, mainly in India, Brazil, and East Africa [7]. In 2023, Brazil accounted for 91% of cases in the Americas [8].

Although incidence has stabilized or declined in some regions in recent years, VL lethality remains a major concern. In Brazil, the case-fatality rate increased by 73.45% between 2008 and 2022 [9] particularly in areas of recent disease emergence and where access to timely diagnosis and treatment is limited [10]. Once considered a zoonosis restricted to rural environments, VL is currently established in urban and peri-urban settings [11], and remains endemic in 24 of Brazil’s 27 states [8]. The spatial and temporal distribution of human VL cases across the country is therefore highly heterogeneous [12,13].

As a vector-borne disease (VBD), VL is influenced by interactions among climatic, environmental, and socioeconomic factors [14,15]. Its vectors, phlebotomine sand flies (Diptera: Psychodidae), develop in moist, organic matter-rich environments. Precipitation is essential for their survival, while temperature regulates their life cycle duration [14], as well as the length of the gonotrophic cycle and extrinsic incubation period [16]. Environmental changes, such as deforestation and urban expansion, directly affect the diversity of vertebrate hosts and sand fly populations in Brazil, ultimately influencing the risk of human infection by *Leishmania (Leishmania) infantum* [17,18].

In addition to environmental variables (e.g., vegetation cover, land use, and climatic conditions) that have been linked to VL incidence [12,17,19–21], biological, social, and programmatic vulnerabilities associated with adverse socioeconomic conditions also play a major role in disease occurrence [13,21–29]. Studying VL distribution in Brazil is particularly challenging due to the country’s vast territory and ecological diversity, with each ecoregion exhibiting distinct climatic, ecological, and cultural features. The six major Brazilian biomes strongly influence the distribution of vectors and reservoirs [30,31], and likely the incidence of the disease itself.

Given this complexity, spatial and temporal exploratory analyses are valuable epidemiological tools for identifying patterns or anomalies in case distribution and their associated factors [32–34]. These approaches support both explanation and/or prediction of disease occurrence [33,34], and can be integrated into regression modeling. In this study, we employed Linear Mixed Models (LMMs), which incorporate temporal and spatial structures through random effects and spatial correlation. This approach enables the identification of locally associated factors (e.g., municipalities or points) over time (e.g., years or days) in relation to disease occurrence [35,36].

Understanding the spatial and temporal patterns of VL can inform more targeted strategies for prevention and control. Therefore, this study aimed to analyze human VL incidence in Brazil between 2008 and 2022 and to assess its associations with climatic, environmental, and social factors at both the national level and within the major Brazilian biomes.

## 2. Methods

### 2.1 Study location and period

**Fig 1:**
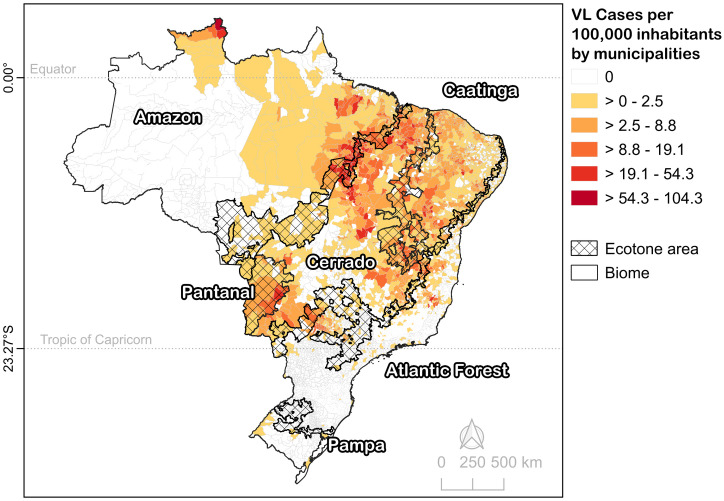
Visceral Leishmaniasis incidence in Brazilian municipalities (2008-2022), including transition of biome boundaries (ecotone areas). Base map: “Biomas_250mil”, IBGE (Brazilian Institute of Geography and Statistics), available at https://www.ibge.gov.br.

Brazil covers 8,510,000 km² and comprises 5,569 municipalities (excluding the island district of Fernando de Noronha). The country is divided into six biomes based on vegetation characteristics: Amazon, Caatinga, Cerrado, Atlantic Forest, Pampa, and Pantanal [31]. Approximately 17.06% (950) of municipalities are located in biome transition zones, referred to as ecotones. The VL cases per inhabitant are heterogeneously distributed across these biomes (Fig 1).

Analyses using Global and Local Spatial Association Indicators and Linear Mixed Models were conducted at both the national and biome-specific levels, including municipalities within ecotones. Biome-specific models were developed only for regions with sufficient epidemiological relevance for VL. The Pampa biome was excluded due to its low proportion of affected municipalities (3.51%; 8/228). Although the Pantanal biome showed a high proportion of municipalities reporting VL cases (72.12%; 17/22), it was not analyzed as a separate biome because of its small number of spatial units and predominant overlap with the Cerrado and Amazon biomes, which limited analytical robustness (Table A in S1 Text).

The municipality was the unit of analysis. When a model referred to a specific biome, all municipalities located fully or partially within its boundaries were included, even those intersecting with Pampa or Pantanal. This decision was justified by the climatic and environmental similarity of these regions and their shared vectors and hosts. Among the 22 municipalities in the Pantanal, only one was not located in an ecotonal zone, while 15 intersect with the Cerrado and 6 with the Amazon, ensuring that the Pantanal’s epidemiological context was analytically represented.

Biome classification of each municipality was determined in QGIS (v3.22.11) using the intersection function, based on Brazilian Institute of Geography and Statistics (IBGE) shapefiles of biomes and municipal boundaries (2019, public domain data).

Ecotone areas represent transitional zones that share climatic and environmental features of adjoining biomes, influencing vector and host adaptation. However, municipalities within these areas are usually dominated by one biome or another, leading to heterogeneous conditions. Therefore, incorporating ecotones as independent analytical units would increase variability and potentially bias biome-specific associations. By including ecotonal municipalities within their respective biome models, we maintained analytical consistency and ensured representation of the Pantanal region, avoiding redundant modeling and preserving ecological coherence.

### 2.2 Data sources, collection, and processing

#### 2.2.1 Human Visceral Leishmaniasis in Brazil and Biomes.

Human VL case data were extracted from the Notifiable Diseases Information System (SINAN), based on the probable place of infection (municipality), and aggregated by municipality and year (2008–2022). Using census data and annual population estimates [37], we calculated triennial incidence (total cases over three years divided by the mean population for the same period), expressed per 100,000 inhabitants. This measure, referred to as ‘cumulative incidence’ (CI), smooths annual fluctuations in endemic municipalities and allows evaluation of low-endemic areas that may report zero cases in individual years.

#### 2.2.2 Associated factors.

We included potential independent variables with biological plausibility for association with VL (see also Table B in S1 Text), grouped as follows:

a. Environmental variables

Annual Land Use and Land Cover (LULC) data were obtained from MapBiomas – Collection 8.0 [38] and averaged by triennium. The following categories were considered: 1) Forest Formation; 2) Deforestation; 3) Savannah & Grassland area; 4) Agriculture; 5) Agropastoral area; 6) Urban infrastructure; and 7) Urban growth. For each municipality and year, each category was expressed as a percentage of the total municipal area. Altitude (in meters) was initially considered as an environmental variable and was obtained from Alvares *et al*. (2013) [39].

b. Climatic Variables

Temperature (°C) and humidity (%), both mean and range, were obtained from the ERA5 satellite dataset by Copernicus [40]. Data were extracted from raster images covering the entire Brazilian territory at a spatial resolution of 0.25º x 0.25º (~28 km²), using monthly averages for each year. For each municipality, mean raster values were extracted using the Zonal Statistics tool in QGIS. Based on these monthly data, annual means and ranges (difference between maximum and minimum monthly values) were calculated.

c. Social and demographic features

Two indicators described municipal population profiles: 1) Urban population proportion (%), defined as the number of people residing in urban areas divided by the total municipal population; and 2) Urban population density, defined as the number of urban residents divided by the urbanized area (based on extract of MapBiomas data). Urban and rural population were estimated for each triennium, as described below in this section, using data from the 2010 (c10) and 2022 (c22) Brazilian demographic censuses, made publicly available by IBGE [37].

Socioeconomic indicators included the illiteracy index (individuals aged ≥15 years), uncollected waste (%), and unconnected sewage system (%). These indicators were estimated for each triennium, as described below (in this section), by data also obtained from the c10 and c22 [37]. Percentages were computed using IBGE raw data on household waste collection and sewage system coverage per residence in each municipality (Table B in S1 Text).

Because census data were only available for 2010 and 2022, we estimated intermediate values for the remaining triennia. Census values were assigned to the triennia that included their respective years (c10 for 2008–2010, 2009–2011 and 2010–2012; and c22 for 2020–2022). For intermediate triennia, values were interpolated proportionally. For instance, for 2015–2017 (midpoint between censuses), the arithmetic mean of 2010 and 2022 values was used $\left(T_{10:22}=\frac{0.5\times c10+0.5\times c22}{2}\right)$. For asymmetric periods (e.g., 2012–2014), weighted means were calculated according to proximity to census year ($T_{12:14}=\frac{0.8\times c10+0.2\times c22}{2}$).

### 2.3 Descriptive analysis

Annual and triennial VL incidence were analyzed to describe case profiles in each biome. We also compared the number of cases and the number of affected municipalities (≥1 confirmed case) over time.

### 2.4 Global and local spatial association indicators

Spatial autocorrelation analyses were performed to assess the spatial concentration of VL incidence (IncVL), and to evaluate the role of ecotone areas. We applied univariate Global Moran’s I (GMI) and Local Indicators of Spatial Association (LISA) to identify high and low incidence clusters (hotspots and coldspots) and to determine whether these overlapped biome boundaries.

Spatial weights were based on minimum distance centroids, ensuring that all municipalities had at least one neighbor. Correlation strength was inversely proportional to distance. Statistical significance was validated using 999 random permutations to generate pseudo-empirical significance values [41]. All analyses were conducted in GeoDa (v1.14.0).

### 2.5 Linear mixed model (LMM)

The IncVL (dependent variable) was modeled against environmental, climatic, and socioeconomic factors (independent variables) using Linear Mixed Models (LMMs). The dependent variable was the triennial cumulative incidence (CI). Separate models were built for each biome (AmzM: Amazon; CaatM: Caatinga; CerrM: Cerrado; AtlM: Atlantic Forest), as cited in section 2.1, and for the entire country (BrzM), which served as a reference for comparison.

#### 2.5.1 Variables selection.

Independent variables were selected for inclusion in the model through a four-step process: 1) Biological Plausibility: Preselection based on existing evidence of relevance to vector ecology or transmission cycle; 2) Spatial Distribution Mapping: Variables with low or irregular distribution were excluded after QGIS based mapping; 3) Correlation Analysis: Spearman’s correlation (ρ) was applied due to non-normal distribution of IncVL (tested beforehand). Variables significantly correlated with IncVL (p < 0.05) were retained. To avoid multicollinearity, when |ρ| ≥ 0.80 and p < 0.05, only the variable most strongly correlated with IncVL was kept; and 4) Forward Stepwise Selection: Variables were sequentially added by strength of correlation, and were remained in the model if: a) its p-value retained consistent (significant or not); b) its coefficient value varied by less than 20%; and c) its sign (positive or negative) remained unchanged.

Few variables were excluded at the selection process steps, mostly at correlation analysis, due to strong multicollinearity. For example, altitude was not included in the final models because it was inversely correlated with temperature. It is important to note that altitude influences VL distribution, as shown by studies in Brazil [42] and other countries [43,44], due to its inverse association with temperature and the stratification of native vegetation along elevation gradients. However, we chose not to include altitude because it would mask the main underlying drivers (temperature is relevant for vectors), which are more meaningful for interpretation. Additionally, given the wide latitudinal range of Brazil, temperature is also shaped by latitude, meaning that altitude would not exert a consistent effect across the country and could introduce bias.

The normality of IncVL was assessed using the Anderson-Darling test via the *nortest* R package [45]. Once all IncVL distributions were non-normal, a log-transformation (log (1 + incVL)) was applied. The QQ plots generated with the *stats* package in R confirmed the adequacy of this transformation (Fig A in S1 Text) [46]. To standardize scales of independent variables, z-scores (value that indicates how far each observation is from the mean) were computed using the *mutate* and *scale* functions from the *dplyr* package [47].

#### 2.5.2 LMM characteristics and assumptions.

The LMMs incorporated random effects to account for spatial and temporal correlation among municipalities. Both triennium (time) and municipality (location) were treated as random effects, while spatial correlation was modeled using the inverse distance between municipal centroids. The Gaussian family was selected because it provided the best model fit with minimal residuals. Model structure followed adaptations from Dormann *et al*., Guo *et al*. and Sevá *et al*. [19,36,48], as described in Equation 1.


$$Log\left[E\left(Y_{it}\right)\right]=\;\alpha_{i}+\;{\beta X}_{it}+\;\alpha^{d_{ij}}$$ (1)

Where *Log[E(Y*_*it*_*)]* is the log-transformed incVL (*Y*_*it*_) in municipality *i (*where *i = *1, 2, 3, …, 5569*)* during triennium *t (*which *t = *2008–2010, 2009–2011, …, 2020–2022), *a*_*i*_ represents the random intercept for municipality *i* (random effect of space); and *βX*_*it*_ denotes the fixed effects (independent variables) in municipality *i* during year *t*. To control residual spatial correlation, the *α* parameter of d_*ij*_ was calculated to represent the inverse distance between the centroids of municipalities *i* and their neighboring municipalities *j*.

Models were run in R software (v 4.2.1) [46] using MASS package and the *glmmPQL* function [49], as represented below. Statistical significance was set at p < 0.05. Both marginal R² (R²m) and conditional R² (R²c) were calculated to represent the variance explained by fixed effects alone and by the full model (fixed + random effects), respectively (the full analysis script can be seen in the S2 Text).

**Model style for all LMMs (BrzM, CaatM, CerrM, Amz and AtlM) in R program**

mod = glmmPQL(log(1+incVL) ~ generic independent variables,

random = ~ 1|triennium|municipality_id,

corr = corSpatial(form=~jitter(x)+y, type = “exponential”),

family = gaussian, data = tab)

Where: municipality_id = municipality code; x and y = longitude and latitude in meters, respectively; tab = dataset.

#### 2.5.3 Results presentation.

Boxplots were generated to assess distributions of significant variables and statistical differences. Since data were non-normally distributed (Anderson-Darling test [50]), the *Kruskal-Wallis* test [51] was applied, followed by *Dunn’s post hoc* test with *Bonferroni* correction [52]. Boxplots and visualizations were generated using the *ggstatsplot* [53] and *ggplot2* [54] packages in R. Normality tests used *nortest* package [45]. Representative maps were created in QGIS, and some figures in Microsoft Excel.

## 3. Results

### 3.1 Descriptive analyses

Between 2008 and 2022, a total of 40,816 human VL cases were reported in Brazil, with 2,310 out of 5,569 municipalities (41.48%) registering at least one case. The year 2017 recorded the highest number of cases (3,692) and the highest number of affected municipalities (950).

The Cerrado biome accounted for the largest proportion of VL cases (47.41%), followed by the Caatinga (32.76%) (Table 1). Despite its larger territorial extent, the Cerrado showed a lower proportion of affected municipalities (58.01%) compared to the Caatinga (76.82%), indicating a higher spatial concentration of VL in the latter. Both biomes reached their peak number of affected municipalities in 2017 (Table 1). However, the Caatinga recorded its highest case count earlier, in 2014 (1,252 cases). In contrast, the Amazon and Atlantic Forest biomes exhibited the lowest proportions of both cases and affected municipalities.

**Table 1:** Comparison of biomes by number and percentage of affected municipalities and total VL cases.

	Total cases (%)	Municipalities		Highest number of affected municipalities (year)	Highest number of cases (year)
Biome		Affected (%)	Affected/Total in biome*		
Caatinga	32.76	76.82	928/1,208	386 (2017)	1,252 (2014)
Cerrado	47.41	58.01	829/1,429	392 (2017)	1,827 (2017)
Amazon	24.75	55.38	309/558	168 (2018)	974 (2017)
Atlantic Forest	18.76	20.89	643/3,078	214 (2017)	711 (2017)
**Brazil**		**41.48**	**2310/5,569**	**950 (2017)**	**3,692 (2017)**

*Total municipalities per biome include all those partially or entirely within each biome. Affected municipalities had at least one reported case.

Although the Pantanal biome was not included as an individual model analysis, it presented the highest proportion on affected municipalities (77.27%; 17/22), only one of which was located entirely outside ecotone areas. The highest number of affected municipalities occurred in 2013, whereas case counts peaked in 2011, a pattern distinct from that observed in the other biomes.

Brazil has 950 municipalities (17.06%) located in biome transition zones (ecotones). Of these, 420 (44.21%) reported at least one case of VL during the study period, with incidences values reaching up to 55.37 per 100,000 inhabitants. A considerable share of high-incidence municipalities from all biomes was found in ecotone areas (Fig 1).

**Fig 2:**
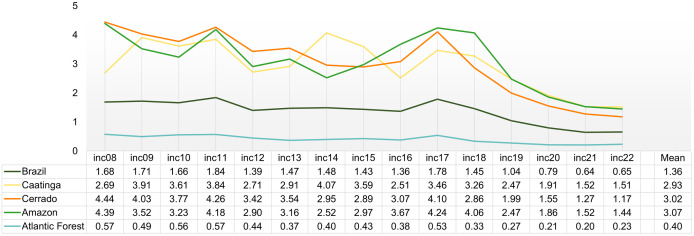
Annual mean incidence (“inc”; per 100,000 inhabitants) in each biome and across Brazil during 2008-2022.

The VL incidence varied widely across biomes and over time. The Caatinga, Cerrado, and Amazon consistently exhibited incidence levels above the national average, whereas the Atlantic Forest presented the lowest values throughout the study period (Fig 2). National annual incidence fluctuated until 2018, after which it declined in all regions. Two main peaks were observed, being one in 2011 and another in 2017. The Cerrado and Amazon followed similar temporal patterns, suggesting a strong influence of shared ecotonal areas. In contrast, the Caatinga displayed a distinct and isolated peak in 2014 (4.07 cases per 100,000).

**Fig 3:**
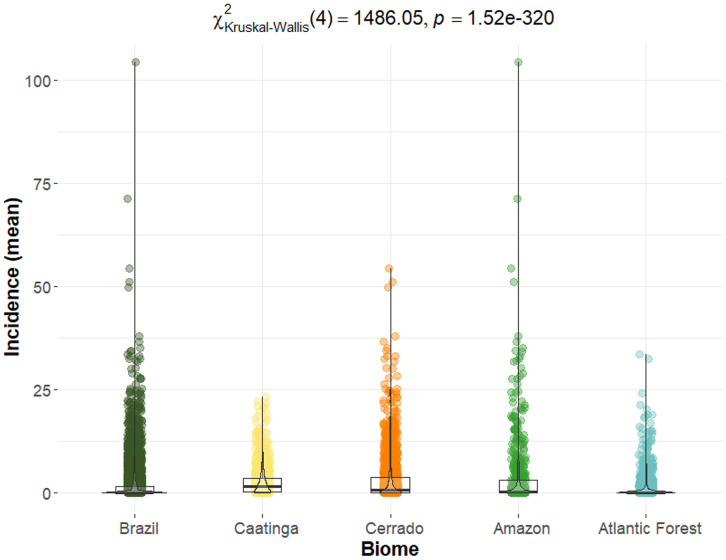
Boxplot of the mean VL incidence (cases per 100,000 inhabitants) by municipality in each biome (2008-2022). Kruskal-Wallis test (p < 0.001) followed by Dunn’s post hoc test indicated significant differences between all biomes, except the Cerrado and Amazon.

Although mean incidence was higher in the Amazon, Cerrado, and Caatinga, these averages were skewed upward by outlier municipalities with very high values, particularly in the Amazon. Dunn’s post hoc test confirmed significant differences in incidence among all biomes, except between the Cerrado and Amazon indicating similar epidemiological profiles between them (Fig 3).

**Fig 4:**
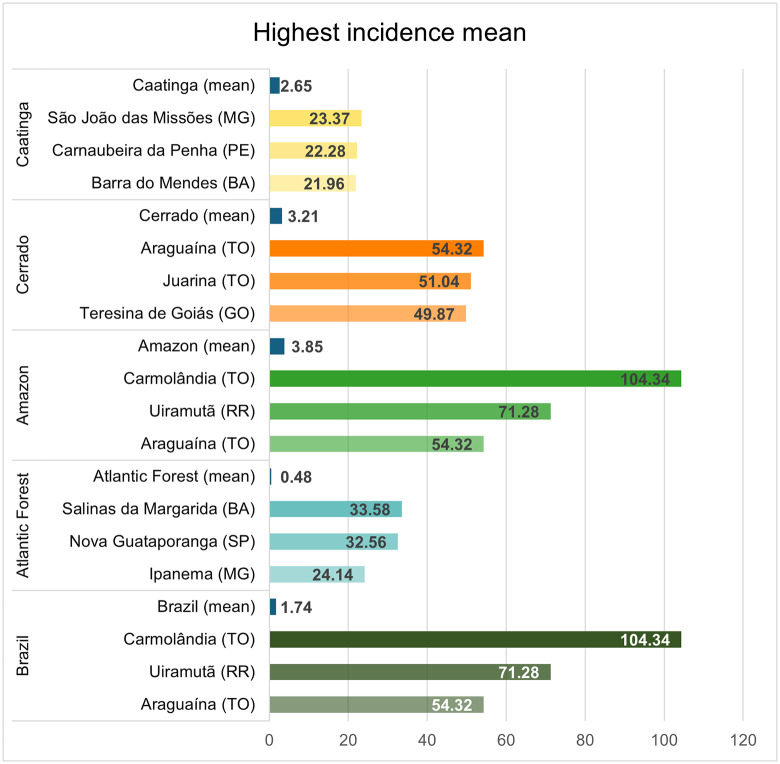
Top three municipalities (state abbreviation) in each biome and nationally with the highest VL incidence (cases per 100,000 inhabitants) over the study period.

Compared with other biomes, the Amazon exhibited a contrasting pattern: despite reporting fewer cases overall (second only to the Atlantic Forest, which had the lowest total), it concentrated the highest incidence outliers in the country, including the three highest national values (Fig 4).

### 3.2 Spatial distribution and Moran’s I analysis

**Fig 5:**
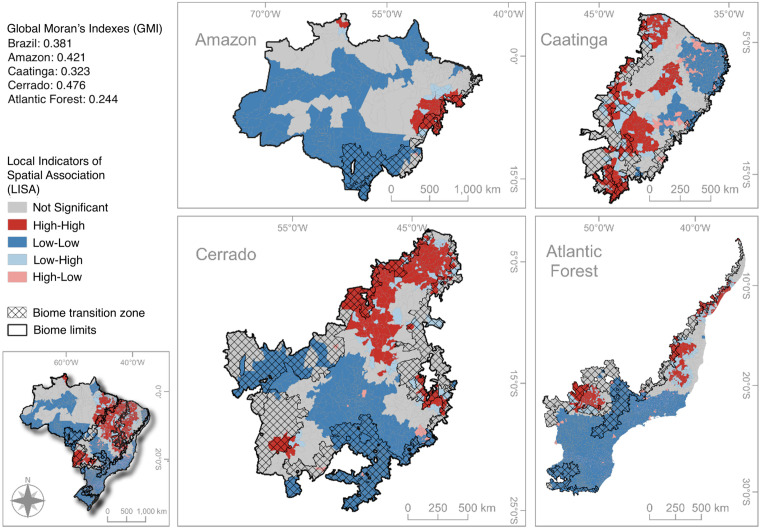
Local Indicators Spatial Autocorrelation (LISA) of VL incidence in Brazil and its biomes. Base map: “Biomas_250mil”, IBGE (Brazilian Institute of Geography and Statistics), available at https://www.ibge.gov.br.

The Local Indicators of Spatial Association (LISA) analysis for Brazil and its biomes revealed significant clusters of high VL incidence (High-High clusters or “hotspots”), especially in the Northeast (Caatinga and Cerrado) and Central-West (Cerrado and Pantanal) regions. Several coldspots were also detected (Fig 5). Ecotone regions exhibited significant clustering, with hotspots particularly evident in Amazon-Cerrado, Cerrado-Caatinga, and Cerrado-Atlantic Forest transitions. Nationwide, a major hotspot was observed in the Cerrado-Pantanal transition zone in the Central West region.

The Cerrado presented the highest Global Moran’s I (GMI) and the largest number of incidence clusters, particularly in the north, overlapping ecotones with the Amazon and Caatinga. The Amazon had the second-highest GMI, with strong clustering near its transition with the Cerrado. The Caatinga displayed a lower GMI but several scattered hotspots, with few clusters in its transition zone with the Atlantic Forest, where coldspots predominated. The Atlantic Forest showed the fewest clusters, restricted mainly to its transitions with the Caatinga and Cerrado. Permutation tests (999 iterations) confirmed that all clustering patterns were statistically significant and non-random.

### 3.3 Comparative analysis of LMMs

The Linear Mixed Models (LMMs) revealed that the factors significantly associated with VL incidence (IncVL) varied across biomes and at the national level, both in terms of presence and direction of association (positive or negative). These findings are summarized in Table 2, with complete model outputs available in Tables C-G in S1 Text. For each biome, only variables meeting the selection criteria (significant correlation and inclusion via forward stepwise selection, as described in the Methods) were retained in the final model.

**Table 2:** Regression coefficient values for each variable in the LMMs across Brazilian biomes and the entire country.

Independent variables	Brazil	Caatinga	Cerrado	Amazon	Atlantic Forest
Agriculture		-0.11	-0.28	-0.26	
Agropastoral areas					
Deforestation	NS	NS	NS	-0.06	
Forest Formation		0.16			NS
Humidity (mean)				-0.18	
Humidity (range)	NS	0.05	NS	NS	
Illiteracy index	-0.04	-0.06			
Savannah & Grassland	0.13			0.17	0.20
Temperature (mean)	0.31			1.17	0.16
Temperature (range)			NS		NS
Uncollected waste	0.02				
Unconnected sewage system	NS	NS		NS	NS
Urban growth		0.03		NS	
Urban infrastructure		NS		NS	NS
Urban population			-0.10		
Urban population density			NS	-0.14	NS

Green: significant positive correlation; Salmon: significant negative correlation: NS: included but not significant; Gray: variables not included in the model after the correlation significance test and forward stepwise selection process.

Although some variables, such as mean temperature, initially demonstrated significant correlations in multiple biomes, their inclusion in the final models depended on covariate interactions and model fit improvements during forward selection process. Thus, a variable could be retained for one biome but excluded from another. To ensure transparency, Table 2 lists all tested variables, including those not retained in specific biome models.

Agriculture appeared in the most biome-specific models and consistently showed a negative correlation with IncVL. Notably, no variable displayed opposite directions of association across biomes (i.e., positive in one and negative in another).

**Fig 6:**
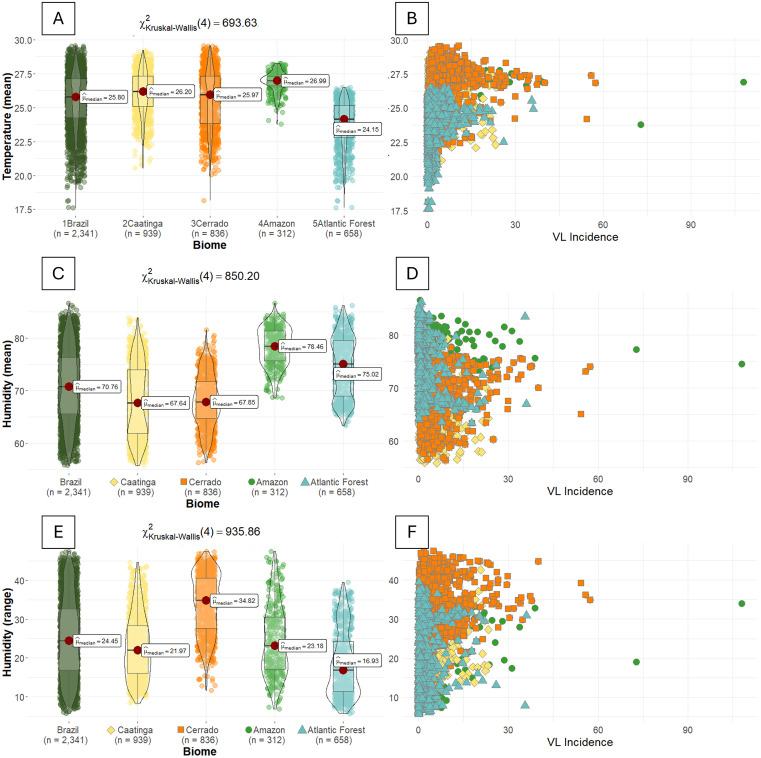
Boxplot (left panels) and scatter plot (right panels) of VL incidence (cases per 100,000 inhabitants) in relation to temperature (mean), humidity (mean and range) in municipalities with at least one VL case in each biome.

The Amazon and Atlantic Forest both showed positive associations between IncVL and savannah & grassland areas, as well as with mean temperature, consistent with the national model. Mean temperature was higher in the Amazon than in the Atlantic Forest (Fig 6A). In the Amazon, IncVL was negatively associated with agriculture, deforestation, mean humidity, and urban population density. Taken together with the positive associations, these findings indicate that VL transmission in the Amazon is concentrated in specific ecological niches, rather than uniformly distributed across all land-use types.

Municipalities classified as outliers (with exceptionally high IncVL values) in both the Amazon and Atlantic Forest also had the highest mean temperatures (Fig 6B). Although it was observed in the Cerrado, this biome did not present significance with the discussed variable. Although temperature range was not significantly associated with IncVL in any biome, the Atlantic Forest displayed the widest thermal range on both mean (Fig 6A) and annual scales (Fig B in S1 Text).

Mean humidity was negatively correlated with IncVL only in the Amazon, which presented the highest median humidity values and the lowest range among all biomes (Fig 6C). Conversely, humidity range was positively associated with IncVL only in the Caatinga biome, which had the widest range of humidity values and the highest temperatures, overlapping with those of the Amazon biome (Fig 6E).

## 4. Discussion

By stratifying the analysis by biome, we observed that ecotone areas played a major role in VL transmission, as several hotspots occurred within these transition zones. Among municipalities reporting at least one VL case during the study period, 17.93% (420/2,342) were located in biome transition. Given their epidemiological importance, characterized by high incidence and overlap with both national and biome-specific hotspots, these municipalities were included in the models of the biomes with which they shared similar environmental features.

The variations among models and the prominent role of ecotones suggest high adaptability of vectors and hosts to changing environments. In these transitional zones, which evolve gradually in space, species with greater environmental tolerance and adaptive plasticity tend to occupy broader ranges and may expand into neighboring ecosystems [55].

Another relevant finding was the variation in associated factors across biome-specific models. Agricultural land was inversely associated with VL incidence in the Caatinga, Cerrado, and Amazon. Interpreting this pattern requires consideration of Brazil’s land ownership structure. In 2017, farms smaller than 50 hectares accounted for 81.40% of agricultural establishments but occupied only 12.80% of the total agricultural land, whereas farms larger than 2,500 hectares represented just 0.30% of establishments but occupied 32.80% of the total area. Land concentration is most pronounced in the Central-West (mainly the Cerrado and parts of the Amazon) [56]. These large farms rely on mechanized agriculture and maintain a low resident population, in contrast to smallholder farms and urban areas.

A study by Afonso *et al*. [17] in Tocantins, a state straddling the Amazon-Cerrado ecotone, also found a negative correlation between agricultural land and sand fly presence. This pattern may reflect the extensive use of pesticides for crop pests control [57], which, combined with low human population density, could reduce VL incidence. However, while fewer VL cases are desirable from a public health perspective, pesticide use raises serious concerns, including birth defects, childhood cancer, human poisoning [57], and broader ecological imbalance. Another possible explanation for the negative correlation is biodiversity loss in monoculture-dominated areas, which can alter vector-hosts interactions and force vectors to seek alternative hosts, including humans [2,58].

The savannah & grassland variable showed a positive association with VL incidence in the Amazon, Atlantic Forest, and national model. High incidence in these biomes occurred mainly in transition zones with the Cerrado, a biome dominated by savannah vegetation [31], which itself accounted for 47.41% of all cases. The Caatinga, which also had a high proportion of affected municipalities and elevated incidence rates, shares key features with the Cerrado, such as open vegetation, absence of dense forests [31], and lower humidity.

Mean temperature was positively associated with VL incidence in the Amazon (interquartile range 25.88ºC - 27.14ºC), Atlantic Forest (19.81ºC - 23.75ºC), and Brazil-wide model, consistent with the known sensitivity of sand fly vectors to climatic variables. Temperature influences vector survival, activity, and parasite development. For instance, in Araguaína (Tocantins), a transition area between the Cerrado and Amazon, nighttime temperatures of 19.90 - 22.70ºC were positively correlated with VL incidence (26.20 cases per 100,000 inhabitants) [59]. Temperatures above 20ºC, combined with low humidity, favor sand fly mobility [60]. This threshold was exceeded in all studied biomes, particularly in the driest biomes, Caatinga and Cerrado.

Despite similar positive associations in the Atlantic Forest and Amazon, median temperatures were lower in the Atlantic Forest and higher in the Amazon (Fig 6). The temperature range was broader in the Atlantic Forest and narrower in the Amazon (Fig B in S1 Text). Both biomes are dominated by dense forest; however, the Amazon model showed inverse associations with IncVL for variables such as humidity, which were not observed in the Atlantic Forest.

Mean humidity was negatively correlated with IncVL only in the Amazon, suggesting that excessively humid and hot conditions may limit vector and/or host activity. Conversely, humidity range was positively correlated with VL incidence in the Caatinga, which is composed predominantly of seasonally dry tropical shrub forests [61,62]. Interestingly, the Caatinga also showed a positive association with forest formation, suggesting that microclimates created by seasonal leaf fall [38] may support vector persistence [63].

This hypothesis may also apply to ecotones between forested biomes and the Cerrado, where deciduous forests prevail due to hydrological stress [64]. A similar dynamic may occur in karst regions of the Cerrado, where savannah vegetation transitions into deciduous dry forest (“Mata Seca”), areas where Ribeiro *et al*. [65] also reported VL hotspots. Future studies should investigate vector occurrence in forest litter within these transitional zones.

Globally, VL vectors tends to prefer thermally stable environments and may be negatively affected by extreme heat and aridity [66]. In the Brazil-wide model, a 1ºC increase in temperature corresponded to a 0.10-unit increase in IncVL. The national mean temperature was 23.52ºC (±3.06ºC), with a wide range (14.90ºC). The VL cases were rare in temperate regions below the Tropic of Capricorn (Fig 1), suggesting that warmer areas drive the observed relationship between temperature and incidence. The Atlantic Forest, which extends into temperate zones, also exhibited a positive temperature-incidence association, with most cases occurring north of the Tropic of Capricorn.

Considering climatic variables, VL incidence was positively associated with mean temperature in the Amazon and Atlantic Forest, and with humidity range in the Caatinga, while a negative association with mean humidity was observed in the Amazon. Under climate change scenarios predominantly driven by global warming [67,68], the positive associations with temperature in the Amazon and Atlantic Forest suggest that areas within these biomes that are not yet climatically favorable may become increasingly suitable for VL transmission. In the Caatinga, increasing humidity range may be intensified by ongoing desertification processes resulting from both anthropogenic and climatic pressures that accentuate the region’s pronounced seasonality [69] More broadly, climate change could make even temperate regions increasingly suitable for the establishment and spread of VL vectors and pathogens. Rising temperatures and altered humidity patters may extend vector activity periods, facilitate adaptation to previously unsuitable areas, and promote southward and altitudinal expansion of transmission risk [66,70,71].

Regarding socioeconomic factors, an unexpected finding was that lower illiteracy rates (i.e., higher literacy) were associated with higher VL incidence in the Caatinga biome and national models. This contrasts with previous studies, which typically report a positive correlation between VL and low socioeconomic indicators, including education level [72]. The illiteracy data used here derived from the IBGE Census and refer to individuals aged ≥ 15 years who reported being unable to read or write a simple note in any language (regardless of schooling history) based on the binary response to the census question “Can you read and write?” [73].

Notably, this variable reflects the entire population in the age group without distinguishing between urban and rural areas, even though VL has become increasingly urban [11,17,22], and illiteracy tends to be more prevalent in rural zones [74]. In the Caatinga, the inverse correlation with illiteracy co-occurred with a positive correlation with urban population, suggesting that urbanization may act as a confounder, masking the direct relationship between education and VL risk.

Other studies have shown positive associations between VL and low education, either independently or as components of broader social vulnerability indices [13,21,22,25–29]. However, those studies often use finer-scale education level indicators, such as years of schooling, rather than municipality-level illiteracy rates. Moreover, self-reported literacy may be biased, as respondents may underreport illiteracy, reinforcing the need for cautious interpretation.

Infrastructure-related factors also influenced VL risk. Uncollected waste was positively associated with incidence in the national model. Accumulated organic matter provides suitable breeding environments for sand fly larvae [27,75,76], underscoring the link between inadequate waste management, environmental conditions, and public health.

Urban growth also showed a positive association with IncVL in the Caatinga, suggesting that expanding urban areas may encroach on vector habitats or that vectors are adapting to peri-urban environments. Conversely, urban population size and density showed negative correlations with VL in the Cerrado and Amazon, respectively. Although urban expansion combined with deforestation can increase human–vector contact [77], deforestation is not always driven by urban growth; it may instead be driven by agriculture, livestock, or logging. In the Amazon, it is primarilly caused by cattle ranching [78]. This nuance is critical when interpreting associations between deforestation and VL risk.

In the Amazon model, urban population density, deforested area, and agricultural land were negatively associated with VL incidence, raising questions about specific transmission settings. Positively associated variables included savannah & grassland vegetation (native *campinarana* formations) and mean temperature, producing a profile similar to high-incidence regions such as the Cerrado and Caatinga. These patterns suggest that VL transmission in the Amazon is more intense in transitional or open vegetation areas than in densely urbanized or intensively farmed landscapes.

Although several variables were significantly associated with VL incidence across Brazil and its biomes, these associations do not imply causality, a limitation inherent to ecological studies based on aggregated municipal-level data. Internal heterogeneity within municipalities, including diverse environmental, social, and climatic conditions, may be masked when averaged. For instance, neighborhoods within the same municipality can differ greatly in urban infrastructure or vegetation cover. Environmental estimates from MapBiomas, derived from 30-meter resolution satellite imagery, may also misclassify complex land use mosaics.

Climatic data from Copernicus were aggregated into annual means and ranges, which may overlook seasonal or short-term variations critical for vector dynamics. Municipal-level microclimates could not be captured due to the national scale of the analysis.

Currently, Brazilian municipalities are classified by endemicity using risk stratification based on VL cases and incidence rates from the previous triennium, which guides prevention and control actions [79]. However, this approach does not account for biome-specific geographic, climatic, or social variability, not for temporal changes driven by biological or anthropogenic factors. More robust risk analyses that incorporate environmental, climatic, and social determinants could enhance understanding of unexpected incidence patterns.

The VL rates in this study were based on SINAN-reported cases. Therefore, underreporting and diagnostic gaps may have led to underestimation of true incidence [80]. Using triennial incidence helped smooth annual fluctuations, especially in low-endemic municipalities where zero-case years may not reflect true absence of transmission. This approach also captured sporadically affected municipalities, reducing underreporting bias. Including random effects by municipality and triennium further adjusted for unobserved heterogeneity.

A reduction in VL incidence was observed in 2017–2018 across all biomes. However, the reasons for this decline remain unclear. Surveillance strategies during this period were limited, and the use of insecticide-impregnated collars for dogs (potentially high-impact intervention) were implemented only after 2020. In addition, the impact of such strategies is expected to occur some time after the implementation. Our models assessed climatic, environmental, and socioeconomic variables over time but did not identify any factor significantly associated with the national-level decline. While some variables may have contributed indirectly, the current analysis does not provide sufficient evidence to explain this trend.

In conclusion, our study demonstrated that climatic, environmental, and social factors influence VL occurrence differently across Brazilian biomes and over time. Ecotonal areas, particularly those between the Amazon and Cerrado, and between the Caatinga and Cerrado, emerged as hotspots, indicating that host-parasite-vector interactions and sandfly populations exhibit remarkable adaptability and ecological flexibility within transition zones. Anthropogenic drivers also contributed substantially to VL dynamics: increasing urban area in the Caatinga, and the presence of savannah and grassland cover within predominantly forested biomes (Amazon and Atlantic Forest), directly influenced the spatial and temporal distribution of cases. In the Caatinga, forested areas were also positively associated with human incidence, whereas larger agricultural areas were associated with lower incidence in the Caatinga, Cerrado, and Amazon.

Municipalities characterized by lower urban population density and percentage or located in more remote areas showed stronger associations with VL, highlighting the importance of rural and peri-urban interfaces. Climatic factors also played a key role: in the Caatinga, a wider humidity range was associated with higher incidence and may be intensified by ongoing desertification, while mean temperature was positively associated with cases in both the Amazon and Atlantic Forest. Only in the Amazon was mean humidity negatively associated with incidence.

From a public health perspective, these findings highlight the need for regionally tailored surveillance and control strategies that account for the ecological specificities of each biome. Strengthening entomological monitoring and integrating environmental and socioeconomic indicators into surveillance systems may improve early detection and support more effective, context-sensitive interventions. Recognizing the spatial heterogeneity of transmission and its associated factors is crucial to sustaining progress toward reducing the burden of VL in Brazil.

## Supporting information

S1 Text **Table A**. Municipalities affected by VL cases from 2008 to 2022 in each biome and in the entire Brazilian territory. **Table B.** Variables, their code, description and source applied in the models of incidence of Visceral Leishmaniasis in humans (incVL) (2008–2022). **Table C.** Correlations of the variables with human VL incidence resulting from the model related to the Caatinga biome (CaatM), along with their respective statistical values. **Table D.** Correlations of the variables with human VL incidence resulting from the model related to the Cerrado biome (CerrM), along with their respective statistical values. **Table E.** Correlations of the variables with human VL incidence resulting from the model related to the Amazon biome (AmzM), along with their respective statistical values. **Table F.** Correlations of the variables with human VL incidence resulting from the model related to the Atlantic Forest biome (AtlM), along with their respective statistical values. **Table G**. Correlations of the variables with human VL incidence resulting from the model related to the Brazil (BrM), along with their respective statistical values. **Fig A**. Quantile-Quantile Plots of Brazil and the studied biomes. **Fig B.** Boxplot (A) and scatter plot (B) of the temperature (range) in municipalities with at least one case in each biome.
(DOCX)

S2 Text R script for statistical analyses. This file contains the complete R script used to perform all statistical analysis described in the manuscript. No figures or tables are included.
(PDF)
