# Supplementary material for: Climatic, environmental, and social factors in Visceral Leishmaniasis: A spatio-temporal perspective in Brazilian biomes
Source: PLoS Negl Trop Dis. 2025 Dec 30;19(12):e0013842. doi: 10.1371/journal.pntd.0013842 (PMC12752973; doi:10.1371/journal.pntd.0013842)
Supplement: S1 Text — Table A. Municipalities affected by VL cases from 2008 to 2022 in each biome and in the entire Brazilian territory. Table B. Variables, their code, description and source applied in the models of incidence of Visceral Leishmaniasis in humans (incVL) (2008–2022). Table C. Correlations of the variables with human VL incidence resulting from the model related to the Caatinga biome (CaatM), along with their respective statistical values. Table D. Correlations of the variables with human VL incidence resulting from the model related to the Cerrado biome (CerrM), along with their respective statistical values. Table E. Correlations of the variables with human VL incidence resulting from the model related to the Amazon biome (AmzM), along with their respective statistical values. Table F. Correlations of the variables with human VL incidence resulting from the model related to the Atlantic Forest biome (AtlM), along with their respective statistical values. Table G. Correlations of the variables with human VL incidence resulting from the model related to the Brazil (BrM), along with their respective statistical values. Fig A. Quantile-Quantile Plots of Brazil and the studied biomes. Fig B. Boxplot (A) and scatter plot (B) of the temperature (range) in municipalities with at least one case in each biome. (DOCX) [file pntd.0013842.s001.docx]

“Climatic, Environmental, and Social Factors in Visceral Leishmaniasis: A Spatio-Temporal Perspective in Brazilian Biomes”

Maíra G. Kersul^1*^, Lucas Edel Donato^2,3^, Alexandre Guerra dos Santos^4^, Rafaella Albuquerque e Silva^2,3^, Marcia Leite de Sousa-Gomes^2^, Fredy Galvis-Ovallos^5^, Vitor Vieira Vasconcelos^6^, Anaiá da Paixão Sevá^1^

¹ Postgraduate Program in Animal Science, State University of Santa Cruz, Ilhéus, Bahia, Brazil

² Health and Environment Surveillance Secretariat / Ministry of Health, Brasília, Federal District, Brazil

³ University Center of Brasília – UNICEUB, Brasília, Federal District, Brazil

⁴ Independent Veterinary Doctor, São Paulo, Brazil

⁵ School of Public Health, University of São Paulo – USP, São Paulo, Brazil

⁶ Center for Engineering, Modeling and Applied Social Sciences – Federal University of ABC, Santo André, São Paulo, Brazil

*Corresponding author

E-mail: [mgkersul@uesc.br](mailto:mgkersul@uesc.br) (MGK)

**Table A**. Municipalities affected by VL cases from 2008 to 2022 in each biome and in the entire Brazilian territory.

|  | **Municipalities** | |
| --- | --- | --- |
| **Biome** | **Affected (%)** | **Affected / Total at Biomas *** |
| Amazon | 56.09 | 313/558 |
| Caatinga | 77.81 | 940/1,208 |
| Cerrado | 58.57 | 837/1,429 |
| Atlantic Forest | 21.38 | 658/3,078 |
| Pampa | 3.51 | 8/228 |
| Pantanal | 77.27 | 17/22 |
| **Brazil** | **42.05** | **2,342/5,569** |

The total number of municipalities in each biome accounts for all those partially or entirely located within its boundaries.

**Table B.** Variables, their code, description and source applied in the models of incidence of Visceral Leishmaniasis in humans (incVL) (2008-2022)

| Variable | Code | Description | Source |
| --- | --- | --- | --- |
| LULC (%) |  |  | Mapbiomas 8.0 |
| Forest Formation | flof |  |  |
| Deforestation | desflof | $\frac{100\times((F_{t}-\left( F_{t-1} \right))}{F_{t-1}}$ |  |
| Savannah & Grassland | savcamp |  |  |
| Agriculture | crop |  |  |
| Agropastoral areas | agrpst | Pasture + Mosaic of Uses |  |
| Urban infrastructure | urban | Urban area(%) + Other non-vegetated area (%) |  |
| Urban growth | vurb | $\frac{100\times((U_{t}-\left( U_{t-1} \right))}{U_{t-1}}$ |  |
| Climatic factors |  |  | [Copernicus Climate Change Services](https://cds.climate.copernicus.eu/cdsapp#!/dataset/ecv-for-climate-change?tab=overview) (ERA 5) |
| Temperature (mean) (ºC) | tmed | $\frac{\sum{monthly average temperature}_{t0}}{12}$ |  |
| Temperature (range) (ºC) | tampli | ${maximum monthly average temperature}_{t0}-{minimum monthly average temperature}_{t0}$ |  |
| Humidity (mean) (%) | u.med | $\frac{\sum{monthly average humidity}_{t0}}{12}$ |  |
| Humidity (range) (%) | u.ampli | ${maximum monthly average humidity}_{t0}-{minimum monthly average humidity}_{t0}$ |  |
| Population |  |  |  |
| Urban population (%) | popurb |  |  |
| Urban population density | durb |  |  |
| Social |  |  | Census from IBGE 2010 and 2022 |
| Illiteracy index | analf | ≥15 years old |  |
| Public Sanitation |  |  | Census from IBGE 2010 and 2022 |
| Uncollected waste (%) | lxncolt | Proportion of (burned waste in property + buried waste in property + waste dumped in vacant lots, hillsides or public areas + other destinies) |  |
| Unconnected sewage system (%) | sgotn | Proportion of (rudimentary pit or hole + ditch + river, lake, stream or sea + other methods + had no toilet or bathroom) |  |
| Biome proportion in the municipalities (%) |  |  | IBGE (2019) |
| Amazon | amz |  |  |
| Atlantic Forest | matl |  |  |
| Caatinga | caat |  |  |
| Cerrado | cerr |  |  |
| Pampa | pamp |  |  |
| Pantanal | pant |  |  |

“t” is for “triennium”;


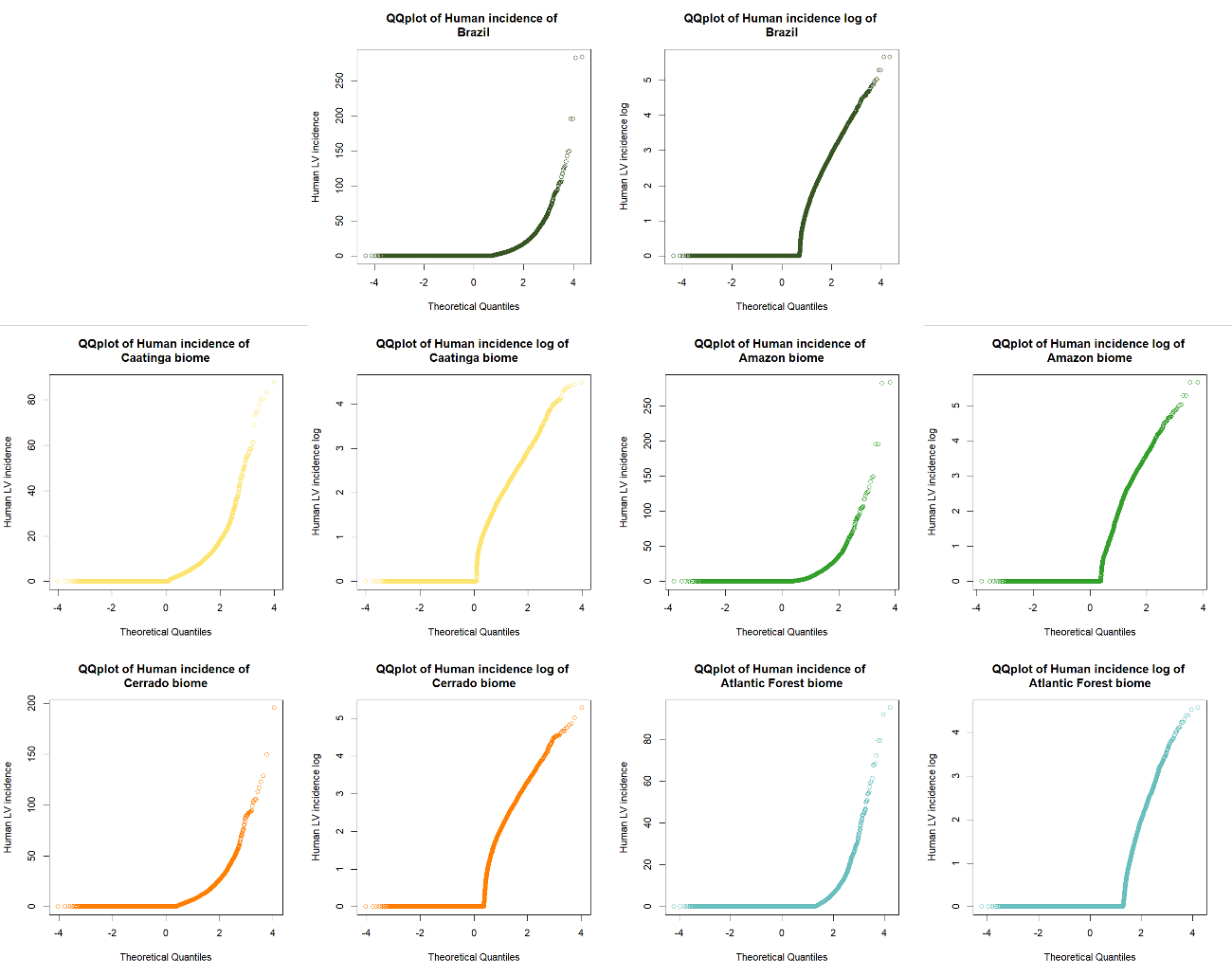


**Fig A. Quantile-Quantile Plots of Brazil and the studied biomes.**

Figure S 2. Comparison of Quantile-Quantile Plots of both human incidence of visceral leishmaniasis (IncVL) and log-transformed of IncVL (log(1+IncVL)) of municipalities of Brazil and the studied biomes isolated.

**Table C.** Correlations of the variables with human VL incidence resulting from the model related to the Caatinga biome (CaatM), along with their respective statistical values.

|  | | **Value** | | **Std.Error** | | **DF** | | **t-value** | | **p-value** | |  |
| --- | --- | --- | --- | --- | --- | --- | --- | --- | --- | --- | --- | --- |
| (Intercept) | | 0.8841 | | 0.0441 | | 14488 | | 20.0371 | | 0 | |  |
| Humidity (range) | | 0.0525 | | 0.0161 | | 14488 | | 3.2540 | | 0.0011 | |  |
| Forest Formation | | 0.1607 | | 0.0371 | | 14488 | | 4.3343 | | <0.001 | |  |
| Illiteracy index | | -0.0623 | | 0.0163 | | 14488 | | -3.8213 | | 0.0001 | |  |
| Agriculture | | -0.1129 | | 0.0516 | | 14488 | | -2.1889 | | 0.0286 | |  |
| Unconnected sewage system | | 0.0068 | | 0.0190 | | 14488 | | 0.3606 | | 0.7184 | |  |
| Urban growth | | 0.0245 | | 0.0055 | | 14488 | | 4.4689 | | <0.001 | |  |
| Urban infrastructure | | -0.0190 | | 0.0359 | | 14488 | | -0.5294 | | 0.5965 | |  |
| Deforestation | | 0.0022 | | 0.0034 | | 14488 | | 0.6597 | | 0.5095 | |  |
| **R²m** |  | |  | |  | |  | |  | | **0.018** | |
| **R²c** |  | |  | |  | |  | |  | | **0.344** | |

DF: Degrees of Freedom; marginal Nakagawa's R² for fixed effects; R²c = conditional Nakagawa's R² for both random and fixed effects

**Table D.** Correlations of the variables with human VL incidence resulting from the model related to the Cerrado biome (CerrM), along with their respective statistical values.

|  | **Value** | **Std.Error** | **DF** | **t-value** | **p-value** |
| --- | --- | --- | --- | --- | --- |
| (Intercept) | 0.6876 | 0.0316 | 17142 | 21.7754 | <0.001 |
| Agriculture | -0.2803 | 0.0214 | 17142 | -13.1077 | <0.001 |
| Temperature (range) | -0.0245 | 0.0246 | 17142 | -0.9947 | 0.3199 |
| Urban population | -0.0961 | 0.0157 | 17142 | -6.1184 | <0.001 |
| Deforestation | -0.0122 | 0.0164 | 17142 | -0.7394 | 0.4597 |
| Urban population density | 0.0135 | 0.0356 | 17142 | 0.3787 | 0.7049 |
| Humidity (range) | 0.0092 | 0.0166 | 17142 | 0.5554 | 0.5786 |
| **R²m** |  |  |  |  | **0.093** |
| **R²c** |  |  |  |  | **0.618** |

DF: Degrees of Freedom; marginal Nakagawa's R² for fixed effects; R²c = conditional Nakagawa's R² for both random and fixed effects

**Table E.** Correlations of the variables with human VL incidence resulting from the model related to the Amazon biome (AmzM), along with their respective statistical values.

|  | **Value** | **Std.Error** | **DF** | **t-value** | **p-value** |
| --- | --- | --- | --- | --- | --- |
| (Intercept) | -0.3880 | 0.1466 | 6686 | -2.6455 | 0 |
| Temperature (mean) | 1.1667 | 0.1049 | 6686 | 11.1186 | <0.001 |
| Humidity (mean) | -0.1843 | 0.0547 | 6686 | -3.3671 | 0.0008 |
| Urban infrastructure | 0.1389 | 0.0834 | 6686 | 1.6648 | 0.0960 |
| Urban density | -0.2242 | 0.0351 | 6686 | -6.3934 | <0.001 |
| Agriculture | -0.2608 | 0.0696 | 6686 | -3.7447 | 0.0002 |
| Savannah & Grassland | 0.1691 | 0.0622 | 6686 | 2.7205 | 0.0065 |
| Deforestation | -0.0603 | 0.0248 | 6686 | -2.4279 | 0.0152 |
| Urban growth | -0.0064 | 0.0050 | 6686 | -1.2762 | 0.2019 |
| Humidity (range) | 0.0076 | 0.0319 | 6686 | 0.2387 | 0.8113 |
| Unconnected sewage system | -0.0141 | 0.0259 | 6686 | -0.5460 | 0.5851 |
| **R²m** |  |  |  |  | **0.131** |
| **R²C** |  |  |  |  | **0.699** |

DF: Degrees of Freedom; marginal Nakagawa's R² for fixed effects; R²c = conditional Nakagawa's R² for both random and fixed effects

**Table F.** Correlations of the variables with human VL incidence resulting from the model related to the Atlantic Forest biome (AltM), along with their respective statistical values.

|  | **Value** | **Std.Error** | **DF** | **t-value** | **p-value** |
| --- | --- | --- | --- | --- | --- |
| (Intercept) | 0.3355 | 0.0133 | 36929 | 25.2524 | <0.001 |
| Temperature (mean) | 0.1562 | 0.0098 | 36929 | 16.0158 | <0.001 |
| Temperature (range) | 0.0036 | 0.0079 | 36929 | 0.4587 | 0.6464 |
| Savannah & Grassland | 0.2000 | 0.0186 | 36929 | 10.7765 | <0.001 |
| Forest Formation | -0.0068 | 0.0082 | 36929 | -0.8201 | 0.4122 |
| Urban population density | -0.0061 | 0.0048 | 36929 | -1.2775 | 0.2014 |
| Urban infrastructure | -0.0060 | 0.0053 | 36929 | -1.1319 | 0.2577 |
| Unconnected sewage system | -0.0015 | 0.0048 | 36929 | -0.3131 | 0.7542 |
| **R²m** |  |  |  |  | **0.095** |
| **R²c** |  |  |  |  | **0.518** |

DF: Degrees of Freedom; marginal Nakagawa's R² for fixed effects; R²c = conditional Nakagawa's R² for both random and fixed effects

**Table G.** Correlations of the variables with human VL incidence resulting from the model related to the Brazil (BrM), along with their respective statistical values.

|  | **Value** | **Std.Error** | **DF** | **t-value** | **p-value** |
| --- | --- | --- | --- | --- | --- |
| (Intercept) | 0.4096 | 0.0079 | 66821 | 51.5640 | <0.001 |
| Temperature (mean) | 0.3095 | 0.0087 | 66821 | 35.4446 | <0.001 |
| Savannah & Grassland | 0.1275 | 0.0089 | 66821 | 14.3808 | <0.001 |
| Illiteracy index | -0.0360 | 0.0080 | 66821 | -4.4897 | <0.001 |
| Humidity (range) | 0.0020 | 0.0063 | 66821 | 0.3199 | 0.7491 |
| Uncollected Waste | 0.0171 | 0.0055 | 66821 | 3.1279 | 0.0018 |
| Unconnected sewage system | -0.0018 | 0.0061 | 66821 | -0.2958 | 0.7674 |
| Deforestation | -0.0004 | 0.0024 | 66821 | -0.1750 | 0.8611 |
| **R²m** |  |  |  |  | **0.189** |
| **R²c** |  |  |  |  | **0.647** |

DF: Degrees of Freedom; marginal Nakagawa's R² for fixed effects; R²c = conditional Nakagawa's R² for both random and fixed effects


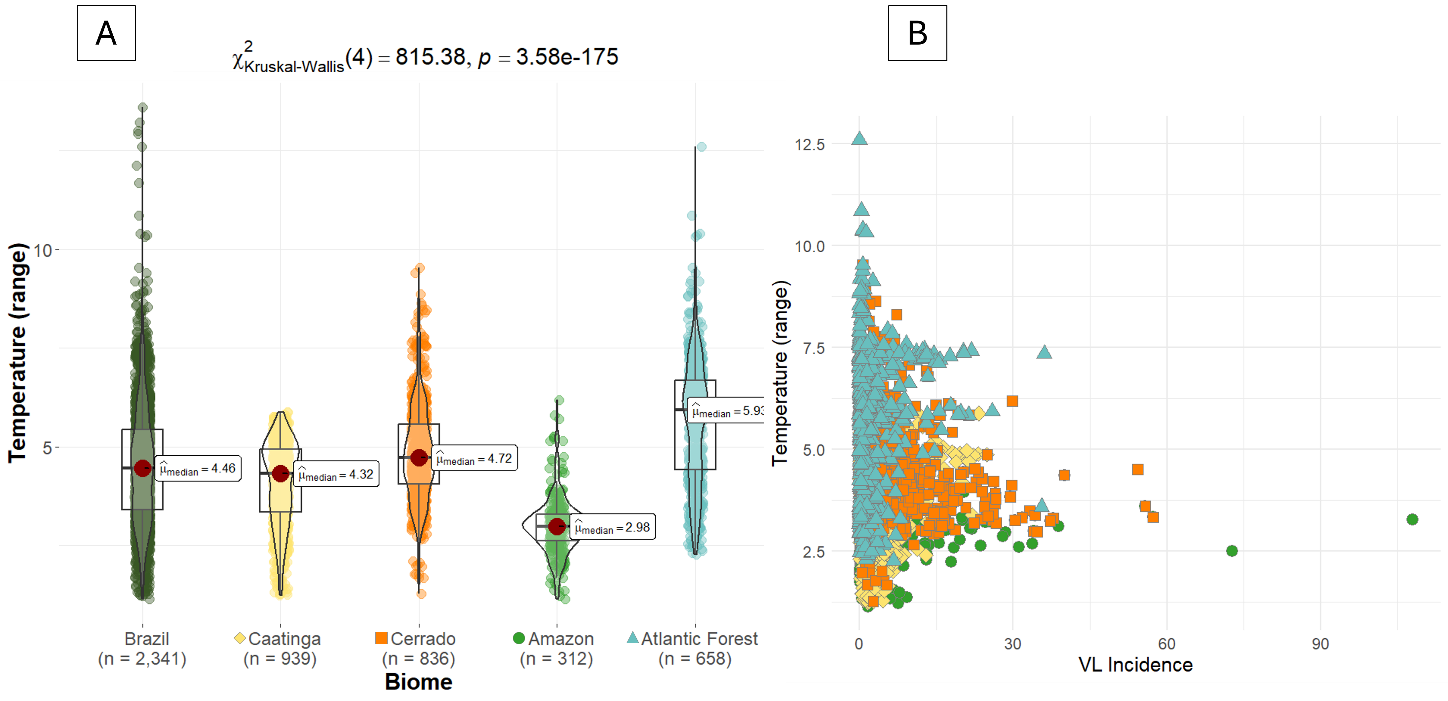


**Fig B. Boxplot (A) and scatter plot (B) of the temperature (range) in municipalities with at least one case in each biome.**
