# Supplementary material for: Climatic, environmental, and social factors in Visceral Leishmaniasis: A spatio-temporal perspective in Brazilian biomes
Source: PLoS Negl Trop Dis. 2025 Dec 30;19(12):e0013842. doi: 10.1371/journal.pntd.0013842 (PMC12752973; doi:10.1371/journal.pntd.0013842)
Supplement: S2 Text — This file contains the complete R script used to perform all statistical analysis described in the manuscript. No figures or tables are included. (PDF) [file pntd.0013842.s002.pdf]

Climatic, Environmental, and Social Factors in Visceral Leishmaniasis: A Spatio-Temporal Perspective in Brazilian Biomes

Maíra G. Kersul and Anaiá da Paixão Sevá

21-jan-2025

Mixed Model for Visceral Leishmaniasis (VL)

Here we present four models for each biome (AmzM: Amazon; CaatM: Caatinga; CerrM: Cerrado; AltM: Atlantic Forest) and one fot the country as a whole (BrzM). The dependent variables are the incidence of VL, and the independent variables are environmental, climatic, social and economic factors, and the random variables are triennium and space (municipalities). The models also considers the distance dependence between municipalities.

Asking for table and fitting it

1. Filtering the correspondent biome

```
library("readxl")
tab <- read_excel("~/MaiKersul(Dout)/tab_VL.xlsx")



#Filter to use each biome

caatbsep <- tab[tab$caat>0,] #Caatinga

cerrbsep <- tab[tab$cerr>0,] #Cerrado

amzbsep <- tab[tab$amz>0,] #Amazon

mtabsep <- tab[tab$matl>0,] #Atlantic Forest
```

Selecting variables to models by evaluating their correlations

The following steps are described in the “Methotds” section “2.5.1” at the manuscript.

We assessed the correlation among variables using the Spearman (ρ) test because of their non-normal distribution.

We selected independent variables that showed a significant correlation ($p<0.05$) with the dependent variable.

For independent variables with strong correlation between each other (ρ > 0.2 or ρ < -0.2 and p < 0.05) there were selected the one most significant with the dependent.

Correlation tests to select variables

1. Evaluating the correlation among all numerical variables by creating a matrix (excluding columns 1 to 5, 8, 9 and 16 to 38 of the variable)
2. Selecting dependent x independent variables p<0.05

```
library("psych") #for correlation matrix

## Brazil (for biomes, just switch "bra" for the correspondent name. For example "caatcorr", for Caatinga)

bracorr <- print(corr.test(tab[,-c(1:5,8,9,16:38)],
                           method = "spearman", adjust = "none",
                           use="pairwise"),
                 short=FALSE)

# Adjusting the table for analysis
bracorr["var_name"] <- row.names(bracorr) #create a column with "variables interactions"
library("stringr")
col <- str_split_fixed(bracorr$var_name, "-", 2) # split the variables in two columns
bracorr <- cbind(bracorr, col)
names(bracorr) [c(8,9)] <- c("var1", "var2") #rename columns with variables
bracorr <- bracorr[,c(2,4,7,8,9)] #create a new dataset with only columns of interetest
depbra <- bracorr[bracorr$var1=="incv",] #create a new dataset with only independent variable correlations
indepbra <- subset(bracorr, var1!="incv") #create a new dataset with only dependent variables correlations

# select dependet x independet variables p<0.05 (column raw.p)
depselectbra <- depbra[depbra$raw.p<0.05,] #select only the significant correlation lines
```

Correlation test of independet variables

Comparison between independet variables.

Identifying the independent variables with high correlation among each other (ρ > 0.2 or ρ < -0.2 and p < 0.05) to then identify the ones that have higher correlation with dependent variable.

```
#to select independet variables with p<0.05 correlations with dependent variable
indepselectbra <- indepbra[indepbra$raw.p<0.05,]
#to select independet variables with rho > 0.2 and rho < -0.2 correlation with independent va
riables
indepselectbra1 <-indepselectbra[indepselectbra$raw.r>0.2,]
indepselectbra2 <-indepselectbra[indepselectbra$raw.r<(-0.2),]

#to unite their results in unique table
indepselectbra <- rbind(indepselectbra1, indepselectbra2)
```

BRAZIL AND BIOMES MODELS

In following these five models, the incidence of VL transformed by log is the dependent variable, and we standardized the independent variables of different scales before inserting in the models

Each model had its independent variables selected as described in the “Methods” section “2.5.1” at the manuscript.

The “random” are random variables, as time in triennium (ano) and municipality code (id6)

Stardardizing the independent variables

```
library("MASS") #for glmmPQL
library("nlme") #for Spatial correlation
library("sjPlot") #for table organization and visualization
library("MuMIn") #for R squared generation

library(dplyr)
tab <- tab %>%
  mutate(
    crop_pad = scale(crop), #agriculture
    agrpst_pad = scale(agrpst), #agropastural areas
    analf_pad = scale(analf), #analphabetism inde
    desflof_pad = scale(desflof), #deforestation
    flof_pad = scale(flof), #forest formation
    u.med_pad = scale(u.med), #humidity (mean)
    u.ampli_pad = scale(u.ampli), #humidity (range)
    savcamp_pad = scale(savcamp), #savannah & grassland
    tmed_pad = scale(tmed), #temperature (mean)
    tampli_pad = scale(tampli), #temperature (range)
    lxncolt_pad = scale(lxncolt), #uncollected waste
    sgotn_pad = scale(sgotn), #unconnected sewage
    urban_pad = scale(urban), #urban infrasturcture
    vurb_pad = scale(vurb), #urban growth
    popurb_pad = scale(popurb), #urban population
    durb_pad = scale(durb) #urban population density
  )

#Filter to use each biome

caatbsep <- tab[tab$caat>0,] #Caatinga

cerrbsep <- tab[tab$cerr>0,] #Cerrado

amzbsep <- tab[tab$amz>0,] #Amazon

mtabsep <- tab[tab$matl>0,] #Atlantic Forest
```

Brazil Model

```
fitbra = glmmPQL(log(1+incv) ~ tmed_pad + savcamp_pad + analf_pad + u.ampli_pad + lxncolt_pad
+ sgotn_pad + desflof_pad,
                 random = ~ 1|ano|id6,
                 corr = corSpatial(form=~jitter(x)+y, type = "exponential"),
                 family = gaussian, data = tab)
summary(fitbra)
```

```
## Linear mixed-effects model fit by maximum likelihood
##   Data: tab
##   AIC BIC logLik
##    NA  NA     NA
##
## Random effects:
##  Formula: ~1 | ano | id6
##  Structure: General positive-definite, Log-Cholesky parametrization
##              StdDev    Corr
## (Intercept) 0.3924021 (Intr)
## 1 | anoTRUE 0.3791768 0.114
## Residual    0.5053076
##
## Correlation Structure: Exponential spatial correlation
##  Formula: ~jitter(x) + y | id6
##  Parameter estimate(s):
##       range
## 5.90508e-06
## Variance function:
##  Structure: fixed weights
##  Formula: ~invwt
## Fixed effects:  log(1 + incv) ~ tmed_pad + savcamp_pad + analf_pad + u.ampli_pad +      lx
ncolt_pad + sgotn_pad + desflof_pad
##                   Value   Std.Error    DF  t-value p-value
## (Intercept)  0.4095584 0.007943086 66821 51.56162  0.0000
## tmed_pad     0.3096766 0.008733081 66821 35.46017  0.0000
## savcamp_pad  0.1275753 0.008869645 66821 14.38337  0.0000
## analf_pad   -0.0361136 0.008031174 66821 -4.49667  0.0000
## u.ampli_pad  0.0018773 0.006307569 66821  0.29763  0.7660
## lxncolt_pad  0.0169782 0.005453878 66821  3.11306  0.0019
## sgotn_pad   -0.0017219 0.006087692 66821 -0.28285  0.7773
## desflof_pad -0.0004206 0.002407216 66821 -0.17474  0.8613
##  Correlation:
##             (Intr) tmd_pd svcmp_ anlf_p u.mpl_ lxncl_ sgtn_p
## tmed_pad     0.000
## savcamp_pad  0.000 -0.271
## analf_pad    0.000 -0.268 -0.183
## u.ampli_pad  0.000 -0.255 -0.174  0.144
## lxncolt_pad  0.000  0.145 -0.021 -0.570 -0.004
## sgotn_pad    0.000 -0.020 -0.012 -0.279 -0.075 -0.143
## desflof_pad  0.000  0.006 -0.037 -0.083  0.045 -0.023  0.016
##
## Standardized Within-Group Residuals:
##         Min          Q1         Med          Q3         Max
## -6.16619392 -0.13464603 -0.03504726  0.06042797  6.28212897
##
## Number of Observations: 72397
## Number of Groups: 5569
```

```
tab_model(fitbra)
```

	log(1 + incv)		
Predictors	*Estimates*	*CI*	*p*

(Intercept)	0.41	0.39 – 0.43	**<0.001**
tmed pad	0.31	0.29 – 0.33	**<0.001**
savcamp pad	0.13	0.11 – 0.14	**<0.001**
analf pad	-0.04	-0.05 – -0.02	**<0.001**
u ampli pad	0.00	-0.01 – 0.01	0.766
lxncolt pad	0.02	0.01 – 0.03	**0.002**
sgotn pad	-0.00	-0.01 – 0.01	0.777
desflof pad	-0.00	-0.01 – 0.00	0.861
**Random Effects**			
σ^2	0.26		
$\tau_{00\ id6}$	0.15		
$\tau_{11\ id6.1\ \mid\ anoTRUE}$	0.14		
$\rho_{01\ id6}$	0.11		
ICC	0.38		
N_{id6}	5569		
Observations	72397		
Marginal R^2 / Conditional R^2	0.250 / 0.532		

```
r.squaredGLMM(fitbra)
```

```
##             R2m       R2c
## [1,] 0.1889132 0.6472011
```

Caatinga Model

```
fitcaatb = glmmPQL(log(1+incv) ~ u.ampli_pad + flof_pad + analf_pad + crop_pad + sgotn_pad +
vurb_pad + urban_pad + desflof_pad,
                random = ~ 1|ano|id6,
                corr = corSpatial(form=~jitter(x)+y, type = "exponential"),
                family = gaussian, data = caatbsep); summary(fitcaatb)
```

```
## Linear mixed-effects model fit by maximum likelihood
##   Data: caatbsep
##   AIC BIC logLik
##    NA  NA     NA
##
## Random effects:
##  Formula: ~1 | ano | id6
##  Structure: General positive-definite, Log-Cholesky parametrization
##              StdDev    Corr
## (Intercept) 0.4840565 (Intr)
## 1 | anoTRUE 0.4829797 -0.11
## Residual    0.6889604
##
## Correlation Structure: Exponential spatial correlation
##  Formula: ~jitter(x) + y | id6
##  Parameter estimate(s):
##        range
## 3.789639e-05
## Variance function:
##  Structure: fixed weights
##  Formula: ~invwt
## Fixed effects:  log(1 + incv) ~ u.ampli_pad + flof_pad + analf_pad + crop_pad +      sgotn
_pad + vurb_pad + urban_pad + desflof_pad
##                  Value  Std.Error    DF   t-value p-value
## (Intercept)  0.8841482 0.04412517 14488 20.037274  0.0000
## u.ampli_pad  0.0524444 0.01612116 14488  3.253143  0.0011
## flof_pad     0.1606925 0.03707712 14488  4.334007  0.0000
## analf_pad   -0.0622710 0.01629155 14488 -3.822286  0.0001
## crop_pad    -0.1128295 0.05155668 14488 -2.188456  0.0287
## sgotn_pad    0.0068342 0.01898149 14488  0.360048  0.7188
## vurb_pad     0.0245112 0.00548300 14488  4.470405  0.0000
## urban_pad   -0.0190049 0.03589164 14488 -0.529508  0.5965
## desflof_pad  0.0022325 0.00338308 14488  0.659889  0.5093
##  Correlation:
##             (Intr) u.mpl_ flf_pd anlf_p crp_pd sgtn_p vrb_pd urbn_p
## u.ampli_pad -0.036
## flof_pad     0.591 -0.001
## analf_pad   -0.163  0.219  0.081
## crop_pad     0.561  0.059 -0.023  0.134
## sgotn_pad   -0.032 -0.055 -0.078 -0.548 -0.039
## vurb_pad    -0.048  0.059  0.021  0.063  0.015  0.015
## urban_pad    0.004  0.102 -0.098  0.128  0.002  0.032  0.036
## desflof_pad  0.040  0.070  0.041 -0.136  0.011  0.019 -0.065  0.001
##
## Standardized Within-Group Residuals:
##         Min          Q1         Med          Q3         Max
## -3.80133160 -0.59286115 -0.08981114  0.52440258  4.06179864
##
## Number of Observations: 15704
## Number of Groups: 1208
```

```
tab_model(fitcaatb)
```

log(1 + incv)

Predictors	*Estimates*	*CI*	*p*
(Intercept)	0.88	0.80 – 0.97	**<0.001**
u ampli pad	0.05	0.02 – 0.08	**0.001**
flof pad	0.16	0.09 – 0.23	**<0.001**
analf pad	-0.06	-0.09 – -0.03	**<0.001**
crop pad	-0.11	-0.21 – -0.01	**0.029**
sgotn pad	0.01	-0.03 – 0.04	0.719
vurb pad	0.02	0.01 – 0.04	**<0.001**
urban pad	-0.02	-0.09 – 0.05	0.596
desflof pad	0.00	-0.00 – 0.01	0.509
**Random Effects**			
σ^2	0.47		
$\tau_{00\ id6}$	0.23		
$\tau_{11\ id6.1\ \mid\ anoTRUE}$	0.23		
$\rho_{01\ id6}$	-0.11		
ICC	0.33		
N $_{id6}$	1208		
Observations	15704		
Marginal R^2 / Conditional R^2	0.018 / 0.342		

```
r.squaredGLMM(fitcaatb)
```

```
##              R2m       R2c
## [1,] 0.01416167 0.4748296
```

Cerrado Model

```
cerrbsep <- transform(cerrbsep, id6 = as.character(id6))
fitcerrb = glmmPQL(log(1+incv) ~ crop_pad + tampli_pad + popurb_pad + desflof_pad + durb_pad
+ u.ampli_pad ,
                   random = ~ 1|ano|id6,
                   corr = corSpatial(form=~jitter(x)+y, type = "exponential"),
                   family = gaussian, data = cerrbsep); summary(fitcerrb)
```

```
## Linear mixed-effects model fit by maximum likelihood
##   Data: cerrbsep
##   AIC BIC logLik
##    NA  NA     NA
##
## Random effects:
##  Formula: ~1 | ano | id6
##  Structure: General positive-definite, Log-Cholesky parametrization
##              StdDev    Corr
## (Intercept) 0.5108590 (Intr)
## 1 | anoTRUE 0.5095603 0.116
## Residual    0.6508985
##
## Correlation Structure: Exponential spatial correlation
##  Formula: ~jitter(x) + y | id6
##  Parameter estimate(s):
##        range
## 1.467115e-05
## Variance function:
##  Structure: fixed weights
##  Formula: ~invwt
## Fixed effects:  log(1 + incv) ~ crop_pad + tampli_pad + popurb_pad + desflof_pad +      du
rb_pad + u.ampli_pad
##                  Value  Std.Error    DF    t-value p-value
## (Intercept)  0.6877652 0.03157806 17142  21.779845  0.0000
## crop_pad    -0.2805105 0.02138299 17142 -13.118395  0.0000
## tampli_pad  -0.0242299 0.02465048 17142  -0.982937  0.3257
## popurb_pad  -0.0963072 0.01570259 17142  -6.133201  0.0000
## desflof_pad -0.0120541 0.01643740 17142  -0.733331  0.4634
## durb_pad     0.0143334 0.03558166 17142   0.402830  0.6871
## u.ampli_pad  0.0095456 0.01663051 17142   0.573980  0.5660
##  Correlation:
##             (Intr) crp_pd tmpl_p pprb_p dsflf_ drb_pd
## crop_pad    -0.018
## tampli_pad   0.260 -0.257
## popurb_pad  -0.268 -0.238 -0.074
## desflof_pad  0.052  0.006  0.049  0.063
## durb_pad     0.434  0.133  0.090 -0.470 -0.106
## u.ampli_pad -0.513  0.096  0.032 -0.088 -0.045  0.102
##
## Standardized Within-Group Residuals:
##         Min          Q1         Med          Q3         Max
## -4.81849781 -0.32364268 -0.05304347  0.12759131  4.76323656
##
## Number of Observations: 18577
## Number of Groups: 1429
```

```
tab_model(fitcerrb)
```

	log(1 + incv)		
Predictors	*Estimates*	*CI*	*p*
(Intercept)	0.69	0.63 – 0.75	**<0.001**

crop pad	-0.28	-0.32 – -0.24	**<0.001**
tampli pad	-0.02	-0.07 – 0.02	0.326
popurb pad	-0.10	-0.13 – -0.07	**<0.001**
desflof pad	-0.01	-0.04 – 0.02	0.463
durb pad	0.01	-0.06 – 0.08	0.687
u ampli pad	0.01	-0.02 – 0.04	0.566
**Random Effects**			
σ^2	0.42		
$\tau_{00\ id6}$	0.26		
$\tau_{11\ id6.1\ \vert\ anoTRUE}$	0.26		
$\rho_{01\ id6}$	0.12		
ICC	0.38		
N_{id6}	1429		
Observations	18577		
Marginal R^2 / Conditional R^2	0.131 / 0.462		

```
r.squaredGLMM(fitcerrb)
```

```
##              R2m       R2c
## [1,] 0.09321898 0.6175884
```

Amazon Model

```
fitamzb = glmmPQL(log(1+incv) ~  tmed_pad + u.med_pad + urban_pad + durb_pad + crop_pad + sav
camp_pad + desflof_pad + vurb_pad + u.ampli_pad + sgotn_pad ,
                  random = ~ 1|ano|id6,
                  corr = corSpatial(form=~jitter(x)+y, type = "exponential"),
                  family = gaussian, data = amzbsep); summary(fitamzb); tab_model(fitamzb)
```

```
## Linear mixed-effects model fit by maximum likelihood
##   Data: amzbsep
##   AIC BIC logLik
##    NA  NA     NA
##
## Random effects:
##  Formula: ~1 | ano | id6
##  Structure: General positive-definite, Log-Cholesky parametrization
##             StdDev    Corr
## (Intercept) 0.4907466 (Intr)
## 1 | anoTRUE 0.5040513 0.272
## Residual    0.5784093
##
## Correlation Structure: Exponential spatial correlation
##  Formula: ~jitter(x) + y | id6
##  Parameter estimate(s):
##        range
## 1.786942e-05
## Variance function:
##  Structure: fixed weights
##  Formula: ~invwt
## Fixed effects:  log(1 + incv) ~ tmed_pad + u.med_pad + urban_pad + durb_pad +      crop_pa
d + savcamp_pad + desflof_pad + vurb_pad + u.ampli_pad +      sgotn_pad
##                  Value  Std.Error   DF   t-value p-value
## (Intercept) -0.3871139 0.14663915 6686 -2.639908  0.0083
## tmed_pad     1.1661681 0.10492635 6686 11.114159  0.0000
## u.med_pad   -0.1858319 0.05476210 6686 -3.393441  0.0007
## urban_pad    0.1380130 0.08340244 6686  1.654784  0.0980
## durb_pad    -0.2233932 0.03507831 6686 -6.368414  0.0000
## crop_pad    -0.2611686 0.06963203 6686 -3.750696  0.0002
## savcamp_pad  0.1688806 0.06214410 6686  2.717565  0.0066
## desflof_pad -0.0605732 0.02484724 6686 -2.437823  0.0148
## vurb_pad    -0.0063897 0.00500380 6686 -1.276971  0.2017
## u.ampli_pad  0.0072710 0.03194869 6686  0.227584  0.8200
## sgotn_pad   -0.0133920 0.02588224 6686 -0.517421  0.6049
##  Correlation:
##             (Intr) tmd_pd u.md_p urbn_p drb_pd crp_pd svcmp_ dsflf_ vrb_pd
## tmed_pad    -0.868
## u.med_pad   -0.664  0.548
## urban_pad    0.106 -0.034  0.020
## durb_pad     0.324 -0.241 -0.050 -0.055
## crop_pad     0.057  0.170  0.234  0.004  0.105
## savcamp_pad  0.051  0.117  0.196  0.018  0.085  0.040
## desflof_pad -0.080  0.083  0.153 -0.010  0.003  0.028  0.065
## vurb_pad     0.000  0.015 -0.009  0.023 -0.141  0.024  0.030  0.015
## u.ampli_pad -0.446  0.310  0.668  0.096  0.051  0.106  0.033  0.107 -0.064
## sgotn_pad   -0.275  0.133  0.068  0.113 -0.345  0.039  0.000 -0.009  0.041
##             u.mpl_
## tmed_pad
## u.med_pad
## urban_pad
## durb_pad
## crop_pad
## savcamp_pad
## desflof_pad
```

```
## vurb_pad
## u.ampli_pad
## sgotn_pad    0.014
##
## Standardized Within-Group Residuals:
##        Min         Q1        Med         Q3        Max
## -5.7391759 -0.3004188 -0.0419177  0.1819343  4.7725680
##
## Number of Observations: 7254
## Number of Groups: 558
```

	**log(1 + incv)**		
Predictors	*Estimates*	*CI*	*p*
(Intercept)	-0.39	-0.67 – -0.10	**0.008**
tmed pad	1.17	0.96 – 1.37	**<0.001**
u med pad	-0.19	-0.29 – -0.08	**0.001**
urban pad	0.14	-0.03 – 0.30	0.098
durb pad	-0.22	-0.29 – -0.15	**<0.001**
crop pad	-0.26	-0.40 – -0.12	**<0.001**
savcamp pad	0.17	0.05 – 0.29	**0.007**
desflof pad	-0.06	-0.11 – -0.01	**0.015**
vurb pad	-0.01	-0.02 – 0.00	0.202
u ampli pad	0.01	-0.06 – 0.07	0.820
sgotn pad	-0.01	-0.06 – 0.04	0.605
**Random Effects**			
σ^2	0.33		
$\tau_{00\ id6}$	0.24		
$\tau_{11\ id6.1\ \mid\ anoTRUE}$	0.25		
$\rho_{01\ id6}$	0.27		
ICC	0.42		
N_{id6}	558		
Observations	7254		
Marginal R^2 / Conditional R^2	0.202 / 0.536		

```
r.squaredGLMM(fitamzb)
```

```
##            R2m       R2c
## [1,] 0.1314226 0.6985108
```

Atlantic Forest Model

```
fitmatlb = glmmPQL(log(1+incv) ~ tmed_pad + tampli_pad + savcamp_pad + flof_pad + durb_pad +
urban_pad + sgotn_pad,
                   random = ~ 1|ano|id6,
                   corr = corSpatial(form=~jitter(x)+y, type = "exponential"),
                   family = gaussian, data = mtabsep); summary(fitmatlb);tab_model(fitmatlb)
```

```
## Linear mixed-effects model fit by maximum likelihood
##   Data: mtabsep
##   AIC BIC logLik
##    NA  NA     NA
##
## Random effects:
##  Formula: ~1 | ano | id6
##  Structure: General positive-definite, Log-Cholesky parametrization
##              StdDev    Corr
## (Intercept) 0.2632438 (Intr)
## 1 | anoTRUE 0.2002168 0.076
## Residual    0.3105922
##
## Correlation Structure: Exponential spatial correlation
##  Formula: ~jitter(x) + y | id6
##  Parameter estimate(s):
##        range
## 0.0003475846
## Variance function:
##  Structure: fixed weights
##  Formula: ~invwt
## Fixed effects:  log(1 + incv) ~ tmed_pad + tampli_pad + savcamp_pad + flof_pad +      durb
_pad + urban_pad + sgotn_pad
##                  Value   Std.Error    DF   t-value p-value
## (Intercept)  0.3355166 0.013287425 36929 25.250689  0.0000
## tmed_pad     0.1561728 0.009752748 36929 16.013211  0.0000
## tampli_pad   0.0036115 0.007893516 36929  0.457525  0.6473
## savcamp_pad  0.2000156 0.018563089 36929 10.774910  0.0000
## flof_pad    -0.0067713 0.008240920 36929 -0.821662  0.4113
## durb_pad    -0.0061153 0.004790595 36929 -1.276532  0.2018
## urban_pad   -0.0059605 0.005266469 36929 -1.131775  0.2577
## sgotn_pad   -0.0014960 0.004798959 36929 -0.311726  0.7553
##  Correlation:
##             (Intr) tmd_pd tmpl_p svcmp_ flf_pd drb_pd urbn_p
## tmed_pad     0.308
## tampli_pad  -0.086  0.527
## savcamp_pad  0.774  0.075  0.030
## flof_pad     0.184  0.410  0.036  0.098
## durb_pad    -0.128 -0.037  0.248  0.012 -0.104
## urban_pad    0.018  0.040  0.001  0.032  0.061 -0.116
## sgotn_pad    0.169  0.095 -0.078 -0.055  0.084 -0.254  0.177
##
## Standardized Within-Group Residuals:
##         Min          Q1         Med          Q3         Max
## -7.97814473 -0.07713389 -0.02711544  0.02904534 10.24059314
##
## Number of Observations: 40014
## Number of Groups: 3078
```

	log(1 + incv)		
Predictors	*Estimates*	*CI*	*p*
(Intercept)	0.34	0.31 – 0.36	**<0.001**

tmed pad	0.16	0.14 – 0.18	**<0.001**
tampli pad	0.00	-0.01 – 0.02	0.647
savcamp pad	0.20	0.16 – 0.24	**<0.001**
flof pad	-0.01	-0.02 – 0.01	0.411
durb pad	-0.01	-0.02 – 0.00	0.202
urban pad	-0.01	-0.02 – 0.00	0.258
sgotn pad	-0.00	-0.01 – 0.01	0.755
**Random Effects**			
σ^2	0.10		
$\tau_{00\ id6}$	0.07		
$\tau_{11\ id6.1\ \mid\ anoTRUE}$	0.04		
$\rho_{01\ id6}$	0.08		
ICC	0.42		
N_{id6}	3078		
Observations	40014		
Marginal R^2 / Conditional R^2	0.103 / 0.478		

```
r.squaredGLMM(fitmatlb)
```

```
##              R2m       R2c
## [1,] 0.08177526 0.5858739
```
